# *p53* Deficiency-Dependent Oncogenicity of Runx3

**DOI:** 10.3390/cells12081122

**Published:** 2023-04-10

**Authors:** Kosei Ito, Shohei Otani, Yuki Date

**Affiliations:** 1 Department of Molecular Bone Biology, Graduate School of Biomedical Sciences, Nagasaki University, 1-7-1 Sakamoto, Nagasaki 852-8588, Japan; 2 Japan Society for the Promotion of Science, 5-3-1 Kojimachi, Chiyoda-ku, Tokyo 102-0083, Japan

**Keywords:** Runx3, p53, c-Myc, osteosarcoma, T-cell lymphoma

## Abstract

The RUNX transcription factors are frequently dysregulated in human cancers, suggesting their potential as attractive targets for drug treatment. However, all three transcription factors have been described as both tumor suppressors and oncogenes, indicating the need to determine their molecular mechanisms of action. Although RUNX3 has long been considered a tumor suppressor in human cancers, several recent studies have shown that RUNX3 is upregulated during the development or progression of various malignant tumors, suggesting it may act as a “conditional” oncogene. Resolving this paradox and understanding how a single gene can exhibit both oncogenic and tumor-suppressive properties is essential for successful drug targeting of RUNX. This review describes the evidence for the activities of RUNX3 in human cancer and proposes an explanation for the duality of RUNX3 involving the status of p53. In this model, *p53* deficiency causes RUNX3 to become oncogenic, leading to aberrant upregulation of MYC.

## 1. Introduction

Three RUNX transcription factors, RUNX1, RUNX2, and RUNX3, along with their cofactor CBFβ, exert tumor-related functions in a context-dependent manner [1]. However, a clear consensus has not yet been reached on the activity of RUNX3, indicating the need for functional analyses. Initially, the gastric phenotype of *Runx3*-knockout mice and the cause-and-effect relationship between loss of RUNX3 and human gastric cancer development suggested that RUNX3 acts as a tumor suppressor [2]. RUNX3 has since been shown to be inactivated by genetic/epigenetic changes [2,3,4,5] or protein mislocalization [6,7,8] in various human cancers, including gastric, colorectal, lung, pancreatic, breast, liver, and prostate cancers, as well as leukemia and neuroblastoma [9]. RUNX3 was originally proposed as a gatekeeper linking oncogenic Wnt and anti-oncogenic TGF-β/BMPs signaling pathways in gastrointestinal tumorigenesis in mice and humans [10]. RUNX3 has also been recognized as an important factor in the regulation of proliferation, differentiation, and apoptosis, as well as in restriction (R)-point, angiogenesis, hypoxic response, epithelial-mesenchymal transition, and DNA repair [9,11,12].

By contrast, RUNX3 was also found to be upregulated in various human malignancies, suggesting that RUNX3 promotes oncogenesis [13]. For example, RUNX3 was shown to enhance tumorigenesis in acute myeloid leukemia [14,15], T-cell acute lymphoblastic lymphoma [16], natural killer/T-cell lymphoma [17], myelodysplastic syndrome [18], skin [19,20], head and neck [21,22], ovarian [23,24,25,26], and pancreatic [27] cancers, and Ewing’s sarcoma [28]. In these tumors, RUNX3 was found to enhance cell proliferation, inhibit apoptosis, and confer drug resistance, indicating that RUNX3 enhances malignant properties associated with the progression of malignancy, such as tumor invasion and metastasis. Most of these studies, however, were unable to determine the precise molecular mechanisms underlying the oncogenic phenotypes observed, although these phenotypes can be attributed to aberrant RUNX3 upregulation.

The ability of RUNX3 to act as both a tumor suppressor gene and an oncogene has indicated that the activity of this gene is dependent on cellular context. Drugs targeting RUNX transcription factors may have clinical value in the cancer treatment [29,30,31], indicating that the determination of RUNX activities is clinically important. Recent osteosarcoma (OS) research has shown that RUNX3 acts as an oncogene by upregulating c-MYC (MYC) in the absence of p53, suggesting that it acts as a tumor suppressor in the presence of intact p53. The cancer-promoting activity of RUNX3 in the absence of wild-type p53 suggests that the RUNX transcription factors may be ideal anti-cancer targets.

## 2. Oncogenic RUNX in the Absence of p53

The ability of Runx3 to act as an oncogene was revealed by a study of OS [32]. The *p53* gene is the most important tumor suppressor gene in the majority of human cancers, and its inactivation and alterations have been widely implicated in tumor development and malignant transformation [33,34,35,36]. OS development is highly dependent on the functional status of p53. *TP53* inactivation is often observed in sporadic OS [32,37,38], and patients with Li-Fraumeni syndrome possessing germline mutations in *TP53* have a high incidence of OS [39,40]. In mice, systemic *p53* deletion is known to cause OS [41], and restrictive deletion of *p53* in osteoprogenitor and mesenchymal stromal cells results in an almost 100% incidence of OS in *Osterix* (*Osx*)/*Sp7*-Cre; *p53*^fl/fl^ mice (herein, *OS* mice), a widely used animal model of human OS [42,43,44].

**Figure 1:**
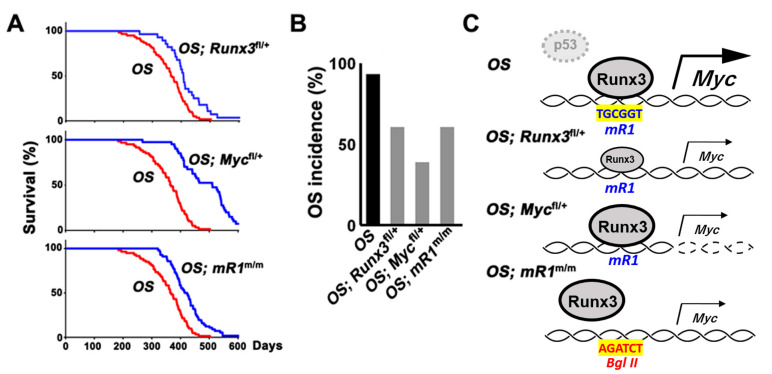
(**A**,**B**) *Osx*-Cre; *p53*^fl/fl^ (*OS*) mice with heterozygous deletions of *Runx3* (*OS*; *Runx3*^fl/+^ mice) or *Myc* (*OS*; *Myc*^fl/+^ mice) or with *mR1* (a Runx consensus site, TGCGGT in the *Myc* promoter) homozygous mutation replaced by the *Bgl II* site, AGATCT (*OS*; *mR1*^m/m^ mice) show a significantly longer life span (**A**) and less incidence of OS development than the original *OS* mice (**B**). (**C**) *Runx3* heterozygous, *Myc* heterozygous, or *mR1* homozygous mutations produce a similar result, i.e., suppression of Myc in vivo. (**A**,**B**) are modified from Otani et al. (2022) [32].

**Figure 2:**
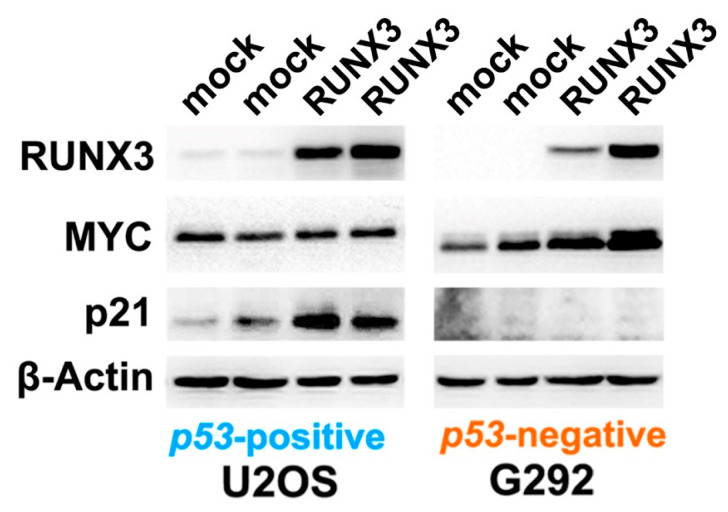
Exogenous RUNX3 upregulates p21 but not MYC in *p53*-positive U2OS cells, but conversely upregulates MYC but not p21 in *p53*-negative G292 cells. The data are modified from Otani et al. (2022) [32].

By using *OS* mice, the root of the tumorigenic process that occurs after p53 inactivation was shown to be Runx3-induced Myc overexpression via *mR1*, a Runx consensus site in the *Myc* promoter [32]. Specifically, both *RUNX3/Runx3* and *MYC/Myc* were upregulated in *p53*-deficient human and mouse OS [32]; heterozygous deletion of *Runx3* (*OS*; *Runx3*^fl/+^ mice) or *Myc* (*OS*; *Myc*^fl/+^ mice) in *OS* mice prolonged their lifespan and suppressed the development of OS (Figure 1A,B). Moreover, in the absence of p53, Runx3 enhanced Myc expression through *mR1*. Therefore, introduction of a homozygous mutation in *mR1* in *OS* mice prolonged their lifespan to the same extent as in *Runx3*-heterozygous *OS* mice, suppressing the development of OS (Figure 1A,B). That is, *Runx3* heterozygosity, *Myc* heterozygosity, and the homozygous *mR1* mutation yielded the same results (Figure 1C). Furthermore, wild-type p53 protein interacted directly with Runx3 protein, inhibiting Runx3 binding to DNA and suppressing Myc overexpression. However, mutants of p53 (R156P and R273H found in human OS cells) that did not interact with RUNX3 were unable to suppress MYC overexpression. Furthermore, suppression of Myc expression by p53 did not occur in cells lacking Runx3, showing that suppression of Myc expression was Runx3-dependent, i.e., p53 directly inhibited Runx3, which has the capacity of upregulating Myc [32]. Although RUNX3 was found to cooperate with p53 to induce p21^WAF1/CIP1^ in *p53*-positive OS (U2OS) cells [45], RUNX3 induced Myc rather than p21^WAF1/CIP1^ in *p53*-negative OS (G292) cells [32] (Figure 2). Thus, the oncogenicity of RUNX3 is dependent on *p53* deficiency during osteosarcomagenesis.

In the presence of p53, however, RUNX3 was found to act as a positive regulator of p53, the gatekeeper and guardian of the genome, during DNA damage and during activation of oncogenes [46,47]. RUNX3 acts as a co-activator for p53, regulating the DNA damage-induced p53 phosphorylation at Ser-15, thereby stabilizing p53 activity and promoting apoptosis [45,48]. RUNX3 is also activated by oncogenic KRAS and indirectly stabilizes p53 by upregulating *p14*^ARF^ (*p19*^Arf^ in mice) in concert with pRB and BRD2, which counters the degradation of p53 by MDM2 [49,50]. Importantly, the tumor suppressor function of RUNX3 appears to be highly dependent on intact p53. Inactivation of p53 is thought to trigger Runx dysregulation, upregulation of Runx3 (and Runx1), and their conversion to oncogenes. Aberrant upregulation of Runx3 has been observed in pancreatic cancer metastases, facilitated in *KPC* mice by loss of heterozygosity (LOH) of *p53* [27,51], and in primary and metastatic gastric cancers, which develop in *Pepsinogen C*-CreER; *Kras*^G12D/+^*Apc*^fl/fl^*p53*^fl/fl^ mice [52]. However, whether Runx3 functions as a driver of metastasis in these *p53*-deficient cancer cells remains to be investigated.

**Figure 3:**
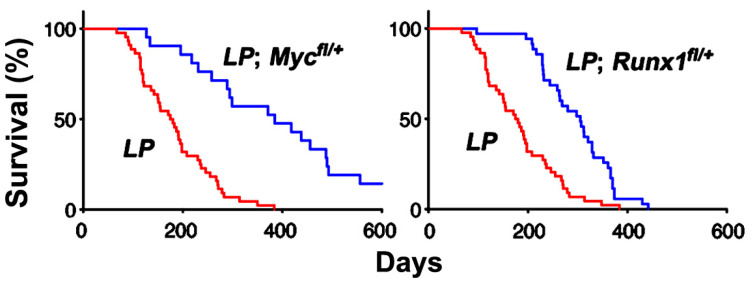
*Lck*-Cre; *p53*^fl/fl^ (*LP*) mice with heterozygous deletions of *Myc* (*LP*; *Myc*^fl/+^ mice) or *Runx1* (*LP*; *Runx1*^fl/+^ mice) show a significantly longer life span than the original *LP* mice. The data are modified from Date et al. (2022) [53].

Runx1 showed similar findings in thymic lymphoma [53], a major tumor type caused by germline *p53* deletion in mice [54]. Deletion of Runx1 was found to suppress T-cell lymphoma development in *p53*-deficient mice [55], and RUNX1 was found to have oncogenic effects on *p53*-null MEFs [56]. Thus, RUNX1 has oncogenic properties in the absence of p53, although RUNX1 also forms a complex with p53 in response to DNA damage and activates the p53 target genes *CDKN1A*, *BAX*, *NOXA,* and *PUMA* [57]. The oncogenic Runx–Myc axis has been reported to play a notable role in mouse thymocytes specifically lacking *p53* (*Lck*-Cre; *p53*^fl/fl^ mouse; herein, *LP* mouse) [53]. Runx1 and Myc are upregulated in *LP* mouse lymphomas, while heterozygous deletions of *Runx1* (*LP*; *Runx1*^fl/+^ mice) or *Myc* (*LP*; *Myc*^fl/+^ mice) prolong the lifespan of these mice (Figure 3) and suppresses lymphoma development. *LP* mice with a homozygous *mR1* mutation have a longer lifespan and a lower incidence of lymphoma [53]. These results, together with the observed oncogenicity of the Runx3-Myc axis in OS development [32], indicate the importance of the RUNX–MYC oncogenic axis acting via *mR1* in the absence of p53. 

RUNX2 was shown to have oncogenic activity in OS [58] and lymphomas [59,60,61], with the loss of p53 and the oncogenic function of RUNX2 being observed in both [58,62,63,64,65]. However, comparative analyses of a recently established *Runx2*-conditional knockout mouse line (*Runx2*-flox) [66] with *Runx1*- and *Runx3*-flox lines showed that Runx2 plays a smaller role as a tumor-promoting factor in *p53*-deficient OS and T-cell lymphoma than do Runx3 and Runx1, respectively [32,53]. Although Runx2 binds p53, its binding activity is weaker than that of Runx3 and Runx1 [32], and unlike Runx3 and Runx1, Runx2 seems to antagonize the tumor-suppressive function of intact p53 [48,67]. These findings suggest that RUNX2 may not be a *p53* deficiency-dependent oncogene. Future studies using improved materials, comprehensive bioinformatics analyses of human tumors, and detailed information obtained using high-throughput NGS analyses may provide more accurate answers.

## 3. RUNX Regulates MYC

Retroviral insertional mutagenesis screens have shown that all three Runx genes act as collaborating oncogenes in Myc-driven lymphoma mouse models [13,68,69,70,71]. These findings were supported by results showing that RUNX and MYC expression are positively correlated in various biological activities [16,17,72,73,74]. In T-cell acute lymphoblastic lymphoma cells, RUNX3 and RUNX1 bind the +1.43 Mb *MYC* enhancer *N-Me* and upregulate MYC expression [16]. In acute myeloid leukemia, however, RUNX1 and its cofactor CBFβ inhibit MYC expression by binding *BDME*, another *MYC* enhancer 0.4 Mb downstream of *N-Me*, indicating that RUNX1 has both tumor-suppressive and oncogenic activities depending on leukemia subtypes [31,75,76]. Thus, the mutual regulation of RUNX and MYC by enhancers/super-enhancers (SEs) for both reveals their close relationship as well as being the basis for their context dependence. It is necessary to identify the SEs responsible for MYC upregulation by RUNX3, especially to determine whether depletion of these genomic elements suppresses tumorigenesis in animal cancer models. The in vivo identification of *mR1* in the *Myc* promoter [32,53] is of great value, showing that Runx positively regulates Myc at its promoter and providing a starting point for a future comprehensive analysis of the positive regulation of *Myc* promoter-SE interactions by Runx, especially Runx3.

RUNX3 has also been shown to prevent tumorigenesis in the gastrointestinal tract, possibly by repressing MYC indirectly. This finding appears to contradict results showing that MYC is activated by RUNX3. In mechanistic terms, RUNX3 attenuates the DNA-binding activity of the β-catenin/TCFs complex that induces MYC, the primary oncogene in gastrointestinal cancer [10,77,78,79]. This tumor-suppressive role of Runx3 was observed in precancerous states using systemic *Runx3*-depleted mouse lines, regardless of p53 status in vivo. In fact, conditional activation of oncogenic Wnt signaling by RUNX3 in gastric cancer cells [10,80] and high expression of Runx3 in *p53*-deficient malignant gastric cancer cells [52] have been reported. Therefore, it is unclear whether Runx3 can continue to function as a tumor suppressor by suppressing Myc after p53 inactivation or whether Runx3 functions as an oncogene by upregulating Myc in these environments. These determinations will require more sophisticated mouse models in which *Runx3* and/or *p53* are disrupted in a tissue- or time-specific manner.

RUNX3 protein is a multiple interactor known to interact with many other transcription factors, which play dual roles in tumorigenesis by integrating oncogenic signals or anti-oncogenic responses [81], such as SMADs in TGF-β signaling [82] and activator protein 1 (AP1) in MAPK signaling [83]. In fact, AP1 transcription factors are prominently upregulated in human and mouse OS, and the consensus motifs of AP1 and RUNX are co-enriched in OS cells, genome-wide [32]. Thus, a contextual determinant of the dual nature of RUNX3 might be affected by other transcription factors in a cancer context. It will be of great interest to determine how RUNX3, released from p53-mediated inhibition of its DNA-binding ability, becomes oncogenic and functions in the upregulation of MYC via its interactions with these transcription factors downstream of cancer-related signals.

## 4. RUNX3 as a Therapeutic Target

Two main types of RUNX inhibitors have been developed and used experimentally. One type comprises the inhibitors AI-10-104 and Ro5-3335, which inhibit the interaction of RUNX with CBFβ [29,84], and the other type comprises pyrrole-imidazole (PI) polyamides, which target the consensus RUNX-binding sequences TGT/CGGT [30]. Inhibition of RUNX by the compound AI-10-104 sensitizes myeloma cell lines and primary tumors to lenalidomide [85] and inhibits the growth of canine OS cells [86]. Combination therapy with Ro5-3335 and SAHA has been reported as a potentially effective way of clearing HIV-1 from cells [87]. Runx site-targeting PI polyamides inhibit the growth of *p53*-negative glioblastoma [88]. Moreover, AI-10-104 and Ro5-3335 have a *p53* deficiency-dependent tumor-suppressive effect on OS and lymphomas in vivo and in vitro [32,53]. However, all of these inhibitors are pan-RUNX inhibitors; thus, a potential obstacle to their clinical application is their potential negative effects on normal RUNXs, although the AI-10-104 dose used in the *p53*-deficient mouse models did not affect appreciably the physiological status of wild-type mice [32]. The development of RUNX species-specific inhibitors, such as middle-molecular compounds that inhibit RUNX3-CBFβ binding, could provide more targeted therapies in the future.

## 5. Conclusions and Perspectives

**Figure 4:**
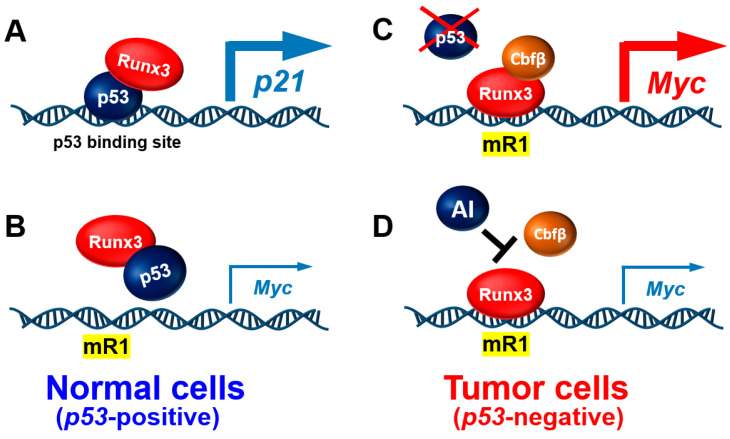
p53 status is the contextual determinant of whether Runx3 functions as a tumor-suppressor or an oncogene. (**A**,**B**) In *p53*-positive normal cells, Runx3, whose transcriptional activation is inhibited by p53, acts as a co-activator of p53 in a tumor-suppressive manner and upregulates p21. (**C**,**D**) In *p53*-negative tumor cells, Runx3 is unleashed from p53 and strongly upregulates Myc as an oncogene. In this context, treatment with a Runx inhibitor (AI-10-104; AI) is effective.

The various findings reported in this review indicated that p53 status is the contextual determinant that determines whether RUNX3 functions as a tumor-suppressor or an oncogene. Accordingly, p53 inactivation would be the key event resulting in the RUNX3 promotion of cancer development. The two major cancer-promoting events, p53 loss and increased Myc signaling, could be linked by Runx3 binding to the mR1 Myc-promoter sequence, which could provide a rationale for the development of RUNX3-targeted therapies against cancer [89]. p53 and MYC have been widely regarded as “undruggable” [90,91], although p53 reactivators exist and have recently entered clinical trials [92]. Rather than directly activating p53 or inhibiting MYC, we suggest that indirectly targeting RUNX3 or *mR1* would provide a more effective alternative for cancer treatment. Since oncogenic transcription by RUNX3 is dormant in *p53*-intact normal cells (Figure 4), if CBFβ is not required for the functional interaction between RUNX3 and p53, the RUNX3-CBFβ interaction is an attractive and widely applicable target for anti-tumor pharmacotherapy in various human cancers and would avoid the side effects of directly targeting RUNX3.

## Data Availability

Not applicable.

## Abbreviations

OS, osteosarcoma; *OS* mouse, *Osterix*/*Sp7*-Cre; *p53*^fl/fl^ mouse, *LP* mouse; *Lck*- Cre, *p53*^fl/fl^ mouse; SE, super-enhancer; AP1, activator protein 1.

## References

[1] Ito Y., Bae S.-C., Chuang L.S.H. (2015). The RUNX family: Developmental regulators in cancer. Nat. Rev. Cancer.

[2] Li Q.L., Ito K., Sakakura C., Fukamachi H., Inoue K.-I., Chi X.-Z., Lee K.-Y., Nomura S., Lee C.-W., Han S.-B. (2002). Causal Relationship between the Loss of RUNX3 Expression and Gastric Cancer. Cell.

[3] Weisenberger D.J., Siegmund K.D., Campan M., Young J., Long T.I., Faasse M.A., Kang G.H., Widschwendter M., Weener D., Buchanan D. (2006). CpG island methylator phenotype underlies sporadic microsatellite instability and is tightly associated with BRAF mutation in colorectal cancer. Nat. Genet..

[4] Chuang L.S.H., Ito Y. (2010). RUNX3 is multifunctional in carcinogenesis of multiple solid tumors. Oncogene.

[5] Douchi D., Yamamura A., Matsuo J., Lee J.-W., Nuttonmanit N., Lim Y.H.M., Suda K., Shimura M., Chen S., Pang S. (2022). A Point Mutation R122C in RUNX3 Promotes the Expansion of Isthmus Stem Cells and Inhibits Their Differentiation in the Stomach. Cell. Mol. Gastroenterol. Hepatol..

[6] Ito K., Liu Q., Salto-Tellez M., Yano T., Tada K., Ida H., Huang C., Shah N., Inoue M., Rajnakova A. (2005). RUNX3, A Novel Tumor Suppressor, Is Frequently Inactivated in Gastric Cancer by Protein Mislocalization. Cancer Res.

[7] Lau Q.C., Raja E., Salto-Tellez M., Liu Q., Ito K., Inoue M., Putti T.C., Loh M., Ko T.K., Huang C. (2006). RUNX3 Is Frequently Inactivated by Dual Mechanisms of Protein Mislocalization and Promoter Hypermethylation in Breast Cancer. Cancer Res.

[8] Goh Y.-M., Cinghu S., Hong E.T.H., Lee Y.-S., Kim J.-H., Jang J.-W., Li Y.-H., Chi X.-Z., Lee K.-S., Wee H. (2010). Src Kinase Phosphorylates RUNX3 at Tyrosine Residues and Localizes the Protein in the Cytoplasm. J. Biol. Chem..

[9] Chuang L.S.H., Ito K., Ito Y. (2017). Roles of RUNX in Solid Tumors. Adv. Exp. Med. Biol..

[10] Ito K. (2011). RUNX3 in oncogenic and anti-oncogenic signaling in gastrointestinal cancers. J. Cell. Biochem..

[11] Chi X.-Z., Lee J.-W., Lee Y.-S., Park I.Y., Ito Y., Bae S.-C. (2017). Runx3 plays a critical role in restriction-point and defense against cellular transformation. Oncogene.

[12] Lee S.H., Manandhar S., Lee Y.M. (2017). Roles of RUNX in Hypoxia-Induced Responses and Angiogenesis. Adv. Exp. Med. Biol..

[13] Neil J.C., Gilroy K., Borland G., Hay J., Terry A., Kilbey A. (2017). The RUNX Genes as Conditional Oncogenes: Insights from Retroviral Targeting and Mouse Models. Adv. Exp. Med. Biol..

[14] Damdinsuren A., Matsushita H., Ito M., Tanaka M., Jin G., Tsukamoto H., Asai S., Ando K., Miyachi H. (2015). FLT3-ITD drives Ara-C resistance in leukemic cells via the induction of RUNX3. Leuk. Res..

[15] Zhang W., Ma Q., Long B., Sun Z., Liu L., Lin D., Zhao M. (2021). Runt-Related Transcription Factor 3 Promotes Acute Myeloid Leukemia Progression. Front. Oncol..

[16] Choi A., Illendula A., Pulikkan J.A., Roderick J.E., Tesell J., Yu J., Hermance N., Zhu L.J., Castilla L.H., Bushweller J.H. (2017). RUNX1 is required for oncogenic Myb and Myc enhancer activity in T-cell acute lymphoblastic leukemia. Blood.

[17] Selvarajan V., Osato M., Nah G.S.S., Yan J., Chung T.-H., Voon D.C.-C., Ito Y., Ham M.F., Salto-Tellez M., Shimizu N. (2017). RUNX3 is oncogenic in natural killer/T-cell lymphoma and is transcriptionally regulated by MYC. Leukemia.

[18] Yokomizo-Nakano T., Kubota S., Bai J., Hamashima A., Morii M., Sun Y., Katagiri S., Iimori M., Kanai A., Tanaka D. (2020). Overexpression of RUNX3 Represses RUNX1 to Drive Transformation of Myelodysplastic Syndrome. Cancer Res.

[19] Salto-Tellez M., Peh B.K., Ito K., Tan S.H., Chong P.Y., Han H.C., Tada K., Ong W.Y., Soong R., Voon D.C. (2006). RUNX3 protein is overexpressed in human basal cell carcinomas. Oncogene.

[20] Lee J.H., Pyon J.-K., Kim D.W., Lee S.H., Nam H.S., Kang S.G., Kim C.H., Lee Y.J., Chun J.S., Cho M.K. (2011). Expression of RUNX3 in skin cancers. Clin. Exp. Dermatol..

[21] Tsunematsu T., Kudo Y., Iizuka S., Ogawa I., Fujita T., Kurihara H., Abiko Y., Takata T. (2009). RUNX3 Has an Oncogenic Role in Head and Neck Cancer. PLoS ONE.

[22] Park J., Kim H.-J., Kim K.R., Lee S.K., Kim H., Park K.-K., Chung W.-Y. (2016). Loss of RUNX3 expression inhibits bone invasion of oral squamous cell carcinoma. Oncotarget.

[23] Barghout S.H., Zepeda N., Vincent K., Azad A.K., Xu Z., Yang C., Steed H., Postovit L.-M., Fu Y. (2015). RUNX3 contributes to carboplatin resistance in epithelial ovarian cancer cells. Gynecol. Oncol..

[24] Lee C.W.L., Chuang L.S.H., Kimura S., Lai S.K., Ong C.W., Yan B., Salto-Tellez M., Choolani M., Ito Y. (2011). RUNX3 functions as an oncogene in ovarian cancer. Gynecol. Oncol..

[25] Chen H., Crosley P., Azad A.K., Gupta N., Gokul N., Xu Z., Weinfeld M., Postovit L.-M., Pangas S.A., Hitt M.M. (2019). RUNX3 Promotes the Tumorigenic Phenotype in KGN, a Human Granulosa Cell Tumor-Derived Cell Line. Int. J. Mol. Sci..

[26] Nevadunsky N.S., Barbieri J.S., Kwong J., Merritt M.A., Welch W.R., Berkowitz R.S., Mok S.C. (2009). RUNX3 protein is overexpressed in human epithelial ovarian cancer. Gynecol. Oncol..

[27] Whittle M.C., Izeradjene K., Rani P.G., Feng L., Carlson M.A., DelGiorno K.E., Wood L.D., Goggins M., Hruban R.H., Chang A.E. (2015). RUNX3 Controls a Metastatic Switch in Pancreatic Ductal Adenocarcinoma. Cell.

[28] Bledsoe K.L., McGee-Lawrence M.E., Camilleri E.T., Wang X., Riester S.M., van Wijnen A.J., Oliveira A.M., Westendorf J.J. (2014). RUNX3 Facilitates Growth of Ewing Sarcoma Cells. J. Cell. Physiol..

[29] Cunningham L., Finckbeiner S., Hyde R.K., Southall N., Marugan J., Yedavalli V.R.K., Dehdashti S.J., Reinhold W.C., Alemu L., Zhao L. (2012). Identification of benzodiazepine Ro5-3335 as an inhibitor of CBF leukemia through quantitative high throughput screen against RUNX1–CBFβ interaction. Proc. Natl. Acad. Sci. USA.

[30] Morita K., Suzuki K., Maeda S., Matsuo A., Mitsuda Y., Tokushige C., Kashiwazaki G., Taniguchi J., Maeda R., Noura M. (2017). Genetic regulation of the RUNX transcription factor family has antitumor effects. J. Clin. Investig..

[31] Bushweller J.H. (2019). Targeting transcription factors in cancer—from undruggable to reality. Nat. Rev. Cancer.

[32] Otani S., Date Y., Ueno T., Ito T., Kajikawa S., Omori K., Taniuchi I., Umeda M., Toguchida J., Ito K. (2021). Runx3 is required for oncogenic Myc upregulation in p53-deficient osteosarcoma. Oncogene.

[33] Baker S.J., Fearon E.R., Nigro J.M., Hamilton S.R., Preisinger A.C., Jessup J.M., Vantuinen P., Ledbetter D.H., Barker D.F., Nakamura Y. (1989). Chromosome 17 Deletions and p53 Gene Mutations in Colorectal Carcinomas. Science.

[34] Hollstein M., Sidransky D., Vogelstein B., Harris C.C. (1991). p53 Mutations in Human Cancers. Science.

[35] Lawrence M.S., Stojanov P., Mermel C.H., Robinson J.T., Garraway L.A., Golub T.R., Meyerson M., Gabriel S.B., Lander E.S., Getz G. (2014). Discovery and saturation analysis of cancer genes across 21 tumour types. Nature.

[36] Bouaoun L., Sonkin D., Ardin M., Hollstein M., Byrnes G., Zavadil J., Olivier M. (2016). *TP53*Variations in Human Cancers: New Lessons from the IARC TP53 Database and Genomics Data. Hum. Mutat..

[37] Kansara M., Teng M.W., Smyth M.J., Thomas D.M. (2014). Translational biology of osteosarcoma. Nat. Rev. Cancer.

[38] Chen X., Bahrami A., Pappo A., Easton J., Dalton J., Hedlund E., Ellison D., Shurtleff S., Wu G., Wei L. (2014). Recurrent Somatic Structural Variations Contribute to Tumorigenesis in Pediatric Osteosarcoma. Cell Rep..

[39] Porter D.E., Holden S.T., Steel C.M., Cohen B.B., Wallace M.R., Reid R. (1992). A Significant Proportion of Patients with Osteo-sarcoma May Belong to Li-Fraumeni Cancer Families. J. Bone Jt. Surgery. Br. Vol..

[40] Bougeard G., Renaux-Petel M., Flaman J.-M., Charbonnier C., Fermey P., Belotti M., Gauthier-Villars M., Stoppa-Lyonnet D., Consolino E., Brugières L. (2015). Revisiting Li-Fraumeni Syndrome From *TP53* Mutation Carriers. J. Clin. Oncol..

[41] Donehower L.A., Harvey M., Slagle B.L., McArthur M.J., Montgomery C.A., Butel J.S., Bradley A. (1992). Mice deficient for p53 are developmentally normal but susceptible to spontaneous tumours. Nature.

[42] Walkley C.R., Qudsi R., Sankaran V.G., Perry J.A., Gostissa M., Roth S.I., Rodda S.J., Snay E., Dunning P., Fahey F.H. (2008). Conditional mouse osteosarcoma, dependent on p53 loss and potentiated by loss of Rb, mimics the human disease. Genes Dev..

[43] Berman S.D., Calo E., Landman A.S., Danielian P.S., Miller E.S., West J.C., Fonhoue B.D., Caron A., Bronson R., Bouxsein M.L. (2008). Metastatic osteosarcoma induced by inactivation of *Rb* and *p53* in the osteoblast lineage. Proc. Natl. Acad. Sci. USA.

[44] Calo E., Quintero-Estades J.A., Danielian P.S., Nedelcu S., Berman S.D., Lees J.A. (2010). Rb regulates fate choice and lineage commitment in vivo. Nature.

[45] Yamada C., Ozaki T., Ando K., Suenaga Y., Inoue K.-I., Ito Y., Okoshi R., Kageyama H., Kimura H., Miyazaki M. (2010). RUNX3 Modulates DNA Damage-mediated Phosphorylation of Tumor Suppressor p53 at Ser-15 and Acts as a Co-activator for p53. J. Biol. Chem..

[46] Bae S.-C., Kolinjivadi A.M., Ito Y. (2018). Functional relationship between p53 and RUNX proteins. J. Mol. Cell Biol..

[47] Lee J.-W., van Wijnen A., Bae S.-C. (2017). RUNX3 and p53: How Two Tumor Suppressors Cooperate Against Oncogenic Ras?. Adv. Exp. Med. Biol..

[48] Ozaki T., Nakagawara A., Nagase H. (2013). RUNX Family Participates in the Regulation of p53-Dependent DNA Damage Response. Int. J. Genom..

[49] Lee Y.-S., Lee J.-W., Jang J.-W., Chi X.-Z., Kim J.-H., Li Y.-H., Kim M.-K., Kim D.-M., Choi B.-S., Kim E.-G. (2013). Runx3 Inactivation Is a Crucial Early Event in the Development of Lung Adenocarcinoma. Cancer Cell.

[50] Lee J.-W., Kim D.-M., Jang J.-W., Park T.-G., Song S.-H., Lee Y.-S., Chi X.-Z., Park I.Y., Hyun J.-W., Ito Y. (2019). RUNX3 regulates cell cycle-dependent chromatin dynamics by functioning as a pioneer factor of the restriction-point. Nat. Commun..

[51] Whittle M.C., Hingorani S.R. (2017). Runx3 and Cell Fate Decision in Pancreas Cancer. Adv. Exp. Med. Biol..

[52] Douchi D., Yamamura A., Matsuo J., Lim Y.H.M., Nuttonmanit N., Shimura M., Suda K., Chen S., Pang S., Kohu K. (2021). Induction of Gastric Cancer by Successive Oncogenic Activation in the Corpus. Gastroenterology.

[53] Date Y., Taniuchi I., Ito K. (2022). Oncogenic Runx1–Myc axis in p53-deficient thymic lymphoma. Gene.

[54] Donehower L.A., Lozano G. (2009). 20 years studying p53 functions in genetically engineered mice. Nat. Rev. Cancer.

[55] Shimizu K., Yamagata K., Kurokawa M., Mizutani S., Tsunematsu Y., Kitabayashi I. (2013). Roles of AML1/RUNX1 in T-cell malignancy induced by loss of p53. Cancer Sci..

[56] Wotton S.F., Blyth K., Kilbey A., Jenkins A., Terry A., Bernardin-Fried F., Friedman A.D., Baxter E.W., Neil J.C., Cameron E.R. (2004). RUNX1 transformation of primary embryonic fibroblasts is revealed in the absence of p53. Oncogene.

[57] Wu D., Ozaki T., Yoshihara Y., Kubo N., Nakagawara A. (2013). Runt-related Transcription Factor 1 (RUNX1) Stimulates Tumor Suppressor p53 Protein in Response to DNA Damage through Complex Formation and Acetylation. J. Biol. Chem..

[58] Martin J.W., Zielenska M., Stein G.S., van Wijnen A.J., Squire J.A. (2010). The Role of RUNX2 in Osteosarcoma Oncogenesis. Sarcoma.

[59] Blyth K., Terry A., Mackay N., Vaillant F., Bell M., Cameron E.R., Neil J.C., Stewart M. (2001). Runx2: A novel oncogenic effector revealed by in vivo complementation and retroviral tagging. Oncogene.

[60] Matthijssens F., Sharma N.D., Nysus M., Nickl C.K., Kang H., Perez D.R., Lintermans B., Van Loocke W., Roels J., Peirs S. (2021). RUNX2 regulates leukemic cell metabolism and chemotaxis in high-risk T cell acute lymphoblastic leukemia. J. Clin. Investig..

[61] Cameron E.R., Blyth K., Hanlon L., Kilbey A., Mackay N., Stewart M., Terry A., Vaillant F., Wotton S., Neil J.C. (2003). The Runx genes as dominant oncogenes. Blood Cells, Mol. Dis..

[62] Blyth K., Terry A., O’Hara M., Baxter E.W., Campbell M., Stewart M., Donehower L.A., Onions D.E., Neil J.C., Cameron E.R. (1995). Synergy between a Human C-Myc Transgene and P53 Null Genotype in Murine Thymic Lymphomas: Contrasting Ef-fects of Homozygous and Heterozygous P53 Loss. Oncogene.

[63] Shin M.H., He Y., Marrogi E., Piperdi S., Ren L., Khanna C., Gorlick R., Liu C., Huang J. (2016). A RUNX2-Mediated Epigenetic Regulation of the Survival of p53 Defective Cancer Cells. PLOS Genet..

[64] van der Deen M., Taipaleenmäki H., Zhang Y., Teplyuk N.M., Gupta A., Cinghu S., Shogren K., Maran A., Yaszemski M.J., Ling L. (2013). MicroRNA-34c Inversely Couples the Biological Functions of the Runt-related Transcription Factor RUNX2 and the Tumor Suppressor p53 in Osteosarcoma. J. Biol. Chem..

[65] He Y., de Castro L.F., Shin M.H., Dubois W., Yang H.H., Jiang S., Mishra P.J., Ren L., Gou H., Lal A. (2015). p53 Loss Increases the Osteogenic Differentiation of Bone Marrow Stromal Cells. STEM CELLS.

[66] Qin X., Jiang Q., Nagano K., Moriishi T., Miyazaki T., Komori H., Ito K., Von Der Mark K., Sakane C., Kaneko H. (2020). Runx2 is essential for the transdifferentiation of chondrocytes into osteoblasts. PLOS Genet..

[67] Ozaki T., Wu D., Sugimoto H., Nagase H., Nakagawara A. (2013). Runt-related transcription factor 2 (RUNX2) inhibits p53-dependent apoptosis through the collaboration with HDAC6 in response to DNA damage. Cell Death Dis..

[68] Mikkers H., Allen J., Knipscheer P., Romeyn L., Hart A., Vink E., Berns A. (2002). High-throughput retroviral tagging to identify components of specific signaling pathways in cancer. Nat. Genet..

[69] Stewart M., MacKay N., Cameron E.R., Neil J.C. (2002). The Common Retroviral Insertion Locus *Dsi1* Maps 30 Kilobases Upstream of the P1 Promoter of the Murine *Runx3*/*Cbfa3*/*Aml2* Gene. J. Virol..

[70] Stewart M., Terry A., Hu M., O’Hara M., Blyth K., Baxter E., Cameron E., Onions D.E., Neil J.C. (1997). Proviral insertions induce the expression of bone-specific isoforms of PEBP2αA (CBFA1): Evidence for a new *myc* collaborating oncogene. Proc. Natl. Acad. Sci. USA.

[71] Wotton S., Stewart M., Blyth K., Vaillant F., Kilbey A., Neil J.C., Cameron E.R. (2002). Proviral Insertion Indicates a Dominant Oncogenic Role for Runx1/AML-1 in T-Cell Lymphoma. Cancer Res..

[72] Kubota S., Tokunaga K., Umezu T., Yokomizo-Nakano T., Sun Y., Oshima M., Tan K.T., Yang H., Kanai A., Iwanaga E. (2019). Author Correction: Lineage-specific RUNX2 super-enhancer activates MYC and promotes the development of blastic plasmacytoid dendritic cell neoplasm. Nat. Commun..

[73] Hosoi H., Niibori-Nambu A., Nah G.S.S., Bahirvani A.G., Mok M.M.H., Sanda T., Kumar A.P., Tenen D.G., Ito Y., Sonoki T. (2021). Super-enhancers for RUNX3 are required for cell proliferation in EBV-infected B cell lines. Gene.

[74] Cohen C.J., Davidson C., Selmi C., Bowness P., Knight J.C., Wordsworth B.P., Vecellio M. (2022). Disruption of c-MYC Binding and Chromosomal Looping Involving Genetic Variants Associated With Ankylosing Spondylitis Upstream of the RUNX3 Promoter. Front. Genet..

[75] Shi J., Whyte W.A., Zepeda-Mendoza C.J., Milazzo J.P., Shen C., Roe J.-S., Minder J.L., Mercan F., Wang E., Eckersley-Maslin M.A. (2013). Role of SWI/SNF in acute leukemia maintenance and enhancer-mediated *Myc* regulation. Genes Dev..

[76] Pulikkan J.A., Hegde M., Ahmad H.M., Belaghzal H., Illendula A., Yu J., O’Hagan K., Ou J., Muller-Tidow C., Wolfe S.A. (2018). CBFβ-SMMHC Inhibition Triggers Apoptosis by Disrupting MYC Chromatin Dynamics in Acute Myeloid Leukemia. Cell.

[77] Ito K., Chuang L.S.H., Ito T., Chang T.L., Fukamachi H., Salto–Tellez M., Ito Y. (2011). Loss of Runx3 Is a Key Event in Inducing Precancerous State of the Stomach. Gastroenterology.

[78] Ito K., Lim A.C.-B., Salto-Tellez M., Motoda L., Osato M., Chuang L.S.H., Lee C.W.L., Voon D.C.-C., Koo J.K.W., Wang H. (2008). RUNX3 Attenuates β-Catenin/T Cell Factors in Intestinal Tumorigenesis. Cancer Cell.

[79] Lee C.W.L., Ito K., Ito Y. (2010). Role of RUNX3 in Bone Morphogenetic Protein Signaling in Colorectal Cancer. Cancer Res.

[80] Ju X., Ishikawa T., Naka K., Ito K., Ito Y., Oshima M. (2014). Context-dependent activation of Wnt signaling by tumor suppressor RUNX 3 in gastric cancer cells. Cancer Sci..

[81] Chuang L.S.H., Ito K., Ito Y. (2013). RUNX family: Regulation and diversification of roles through interacting proteins. Int. J. Cancer.

[82] David C.J., Massagué J. (2018). Contextual determinants of TGFβ action in development, immunity and cancer. Nat. Rev. Mol. Cell Biol..

[83] Eferl R., Wagner E.F. (2003). AP-1: A double-edged sword in tumorigenesis. Nat. Rev. Cancer.

[84] Illendula A., Gilmour J., Grembecka J., Tirumala V.S.S., Boulton A., Kuntimaddi A., Schmidt C., Wang L., Pulikkan J.A., Zong H. (2016). Small Molecule Inhibitor of CBFβ-RUNX Binding for RUNX Transcription Factor Driven Cancers. Ebiomedicine.

[85] Zhou N., Gutierrez-Uzquiza A., Zheng X.Y., Chang R., Vogl D.T., Garfall A.L., Bernabei L., Saraf A., Florens L., Washburn M.P. (2019). RUNX proteins desensitize multiple myeloma to lenalidomide via protecting IKZFs from degradation. Leukemia.

[86] Alegre F., Ormonde A.R., Godinez D.R., Illendula A., Bushweller J.H., Wittenburg L.A. (2019). The interaction between RUNX2 and core binding factor beta as a potential therapeutic target in canine osteosarcoma. Veter- Comp. Oncol..

[87] Klase Z., Yedavalli V.S.R.K., Houzet L., Perkins M., Maldarelli F., Brenchley J., Strebel K., Liu P., Jeang K.-T. (2014). Activation of HIV-1 from Latent Infection via Synergy of RUNX1 Inhibitor Ro5-3335 and SAHA. PLOS Pathog..

[88] Hattori E.Y., Masuda T., Mineharu Y., Mikami M., Terada Y., Matsui Y., Kubota H., Matsuo H., Hirata M., Kataoka T.R. (2022). A RUNX-targeted gene switch-off approach modulates the BIRC5/PIF1-p21 pathway and reduces glioblastoma growth in mice. Commun. Biol..

[89] Date Y., Ito K. (2020). Oncogenic RUNX3: A Link between p53 Deficiency and MYC Dysregulation. Mol. Cells.

[90] Sabapathy K., Lane D. (2017). Therapeutic targeting of p53: All mutants are equal, but some mutants are more equal than others. Nat. Rev. Clin. Oncol..

[91] Mullard A. (2022). Climbing cancer’s MYC mountain. Nat. Rev. Drug Discov..

[92] Bykov V.J.N., Eriksson S.E., Bianchi J., Wiman K.G. (2018). Targeting mutant p53 for efficient cancer therapy. Nat. Rev. Cancer.

